# Suppression of miR-181a attenuates H_2_O_2_-induced death of mesenchymal stem cells by maintaining hexokinase II expression

**DOI:** 10.1186/s40659-015-0036-5

**Published:** 2015-08-18

**Authors:** Seahyoung Lee, Ina Yun, Onju Ham, Se-Yeon Lee, Chang Yeon Lee, Jun-Hee Park, Jiyun Lee, Hyang-Hee Seo, Eunhyun Choi, Ki-Chul Hwang

**Affiliations:** Institute for Bio-Medical Convergence, College of Medicine, Catholic Kwandong University, Gangneung, Gangwon-do 210-701 Republic of Korea; Brain Korea 21 PLUS Project for Medical Science, Yonsei University, Seoul, 120-752 Republic of Korea; Department of Integrated Omics for Biomedical Sciences, Yonsei University, Seoul, 120-752 Republic of Korea

**Keywords:** Hexokinase II, miRNA-181a, Cell death, Mesenchymal stem cell

## Abstract

**Background:**

Low survival rate of transplanted cells compromises the efficacy of cell therapy. Hexokinase II (HKII) is known to have anti-apoptotic activity through its interaction with mitochondria. The objective was to identify miRNAs targeting HKII and investigate whether miRNA-mediated modulation of HKII could improve the survival of mesenchymal stem cells (MSCs) exposed to H_2_O_2_. The expression of HKII in MSCs exposed to H_2_O_2_ was evaluated, and HKII-targeting miRNA was screened based on miRNA-target prediction databases. The effect of H_2_O_2_ on the expression of the selected HKII-targeting miRNA was examined and the effect of modulation of the selected HKII-targeting miRNA using anti-miRNA on H_2_O_2_-induced apoptosis of MSC was evaluated.

**Results:**

H_2_O_2_ (600 μM) induced cell death of MSCs and decreased mitochondrial HKII expression. We have identified miR-181a as a HKII-targeting miRNA and H_2_O_2_ increased the expression of miR-181a in MSCs. Delivery of anti-miR-181a, which neutralizes endogenous miR-181a, significantly attenuated H_2_O_2_-induced decrease of HKII expression and disruption of mitochondrial membrane potential, improving the survival of MSCs exposed to H_2_O_2_.

**Conclusions:**

These findings suggest that H_2_O_2_-induced up-regulation of miR-181a contributes to the cell death of MSCs by down-regulating HKII. Neutralizing miR-181a can be an effective way to prime MSCs for transplantation into ischemic tissues.

## Background

For many decades, ischemic heart diseases such as myocardial infarction (MI) have been a leading cause of death worldwide [1]. Cell therapy using various stem cells is one of the therapeutic approaches to manage such heart disease [2]. However, high death rate of the transplanted cells has hampered the efficacy of stem cell-based cell therapy [3]. One of the major reasons for such low survival of transplanted cells is that the transplanted cells are also immediately exposed to a harsh microenvironment (i.e., low tissue oxygen and nutrients, and increased reactive oxygen species (ROS) in case of ischemic heart disease), which can induce various types of cell death, including apoptosis [4]. Consequently, discovery of alternative means to attenuate or prevent the onset of apoptosis of transplanted cells is of utmost importance for developing effective therapeutic strategy.

One of the molecules known to play an important role in preventing apoptosis is hexokinase II (HKII) [5]. HK family is composed of four different isotypes (1 to 4, or I to IV) and they can initiate the glycolysis pathway where glucose is converted to glucose-6-phosphate [6]. Additionally, HKs are known to have non-enzymatic activities such as anti-apoptotic activity [7]. A previous study reported that myocardial ischemia/reperfusion (I/R) injury decreased the cardiac expression of HKII as well as the binding to mitochondria, and it was associated with decreased cardiac function [8]. Thus, assuming the cell death mechanisms of transplanted cells are similar to those of host cells, maintaining the expression of HKII may improve the survival of transplanted cells. Regarding the regulation of HKII expression, we speculate that microRNAs (miRNA or miR) may be involved. MicroRNAs are approximately 22-nucleotide-long RNAs that attenuate certain gene expression by either inhibiting mRNA translation or degrading mRNAs [9], and studies have demonstrated that miRNAs, such as miR-143 and miR-125a-5p, negatively regulated HKII expression in cancer cells [10–12]. This prompted us to examine possible involvement of HKII-targeting microRNAs (miRNAs) in the death of transplanted cells.

In the present study, we examined the possibility of miRNA-dependent HKII regulation during ROS-induced death of mesenchymal stem cells (MSCs). We identified miR-181a as a HKII-targeting miRNA and demonstrated that its expression increased during ROS-induced apoptosis of MSCs. We further examined the potential of anti-miR-181a as an anti-apoptotic agent for MSCs exposed to ROS in vitro.

## Results

### H_2_O_2_ induced cell death of MSCs and decrease of mitochondrial HKII expression

**Fig. 1:**
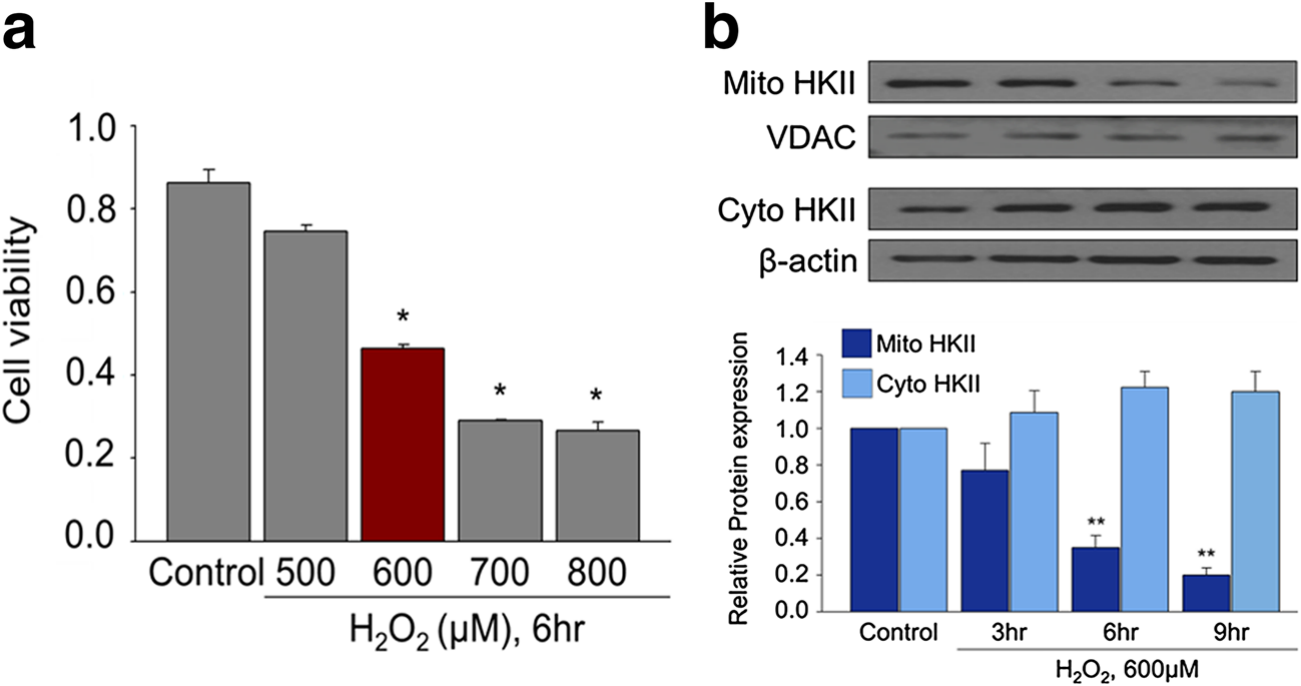
Decrease of mitochondrial HKII during H_2_O_2_-induced MSC cell death. MSCs were exposed to increasing concentrations of H_2_O_2_ simulating ROS for 6 h. **a** Cell viability of MSCs exposed to varying concentrations of H_2_O_2_. *p < 0.05 compared to Control. **b** Time dependent changes of mitochondrial and cytosolic expressions of HKII in MSCs exposed to H_2_O_2_. **p < 0.05 compared to Control. The quantitative data were expressed as the mean ± s.e.m of at least three independent experiments.

H_2_O_2_, which has been used to induce apoptosis of MSCs [13], was used to simulate oxidative stress after transplantation. H_2_O_2_ concentration-dependently decreased cell viability of MSCs, and 600 μM or higher concentrations of H_2_O_2_ significantly decreased cell viability of MSCs after 6 h of treatment (Fig. 1a). Since 6 h of treatment with 600 μM of H_2_O_2_ significantly induced cell death of MSCs, this particular condition was use for further experiments. For the expression of HKII, H_2_O_2_ time-dependently decreased HKII in mitochondrial fraction, while it had no significant effect on HKII in cytosolic fraction (Fig. 1b), suggesting the decrease of mitochondrial HKII contributed to cell death of MSCs exposed to H_2_O_2_.

### Identification of HKII targeting miRNA and its expression during H_2_O_2_-induced cell death

**Fig. 2:**
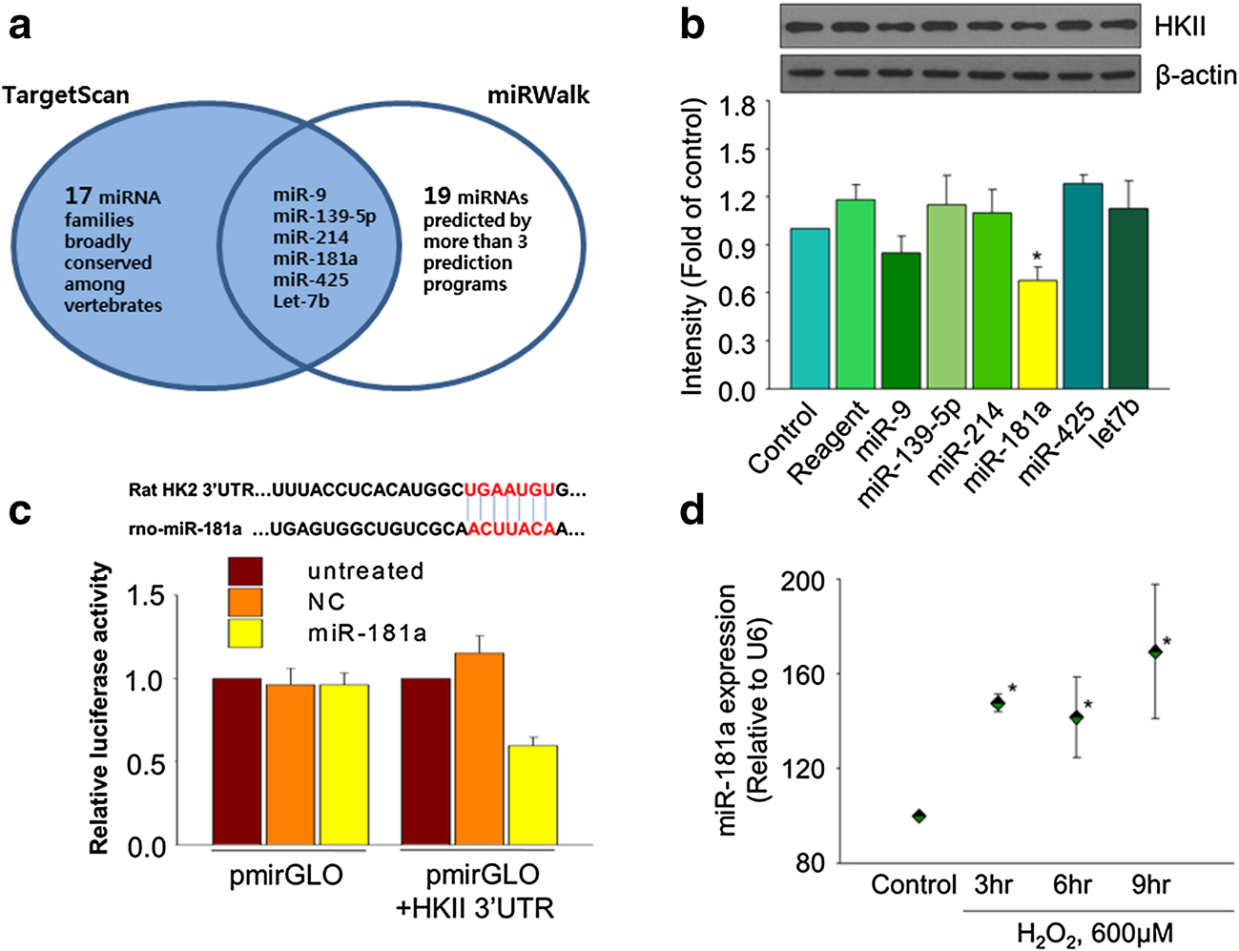
MicroRNA-181a targets HKII and increased by H_2_O_2_. **a** Candidate HKII-targeting miRNAs were selected based on miRNA-target prediction databases (TargetScan and miRWalk). **b** Effect of six candidate miRNAs on the expression of HKII was examined by western blot. *p < 0.05 compared to Control. **c** Luciferase assay using 3′UTR of HKII was performed to confirm the interaction between miR-181a and HKII. *p < 0.05 compared to Control [miR (−)]. *NC* negative control miRNA. **d** Time dependent expression changes of miR-181 in MSCs exposed to H_2_O_2_. *p < 0.05 compared to Control. The quantitative data were expressed as the mean ± s.e.m of at least three independent experiments.

To screen miRNAs targeting HKII, miRNA-target protein prediction databases (http://www.targetscan.org and http://www.umm.uni-heidelberg.de/apps/zmf/mirwalk/) were utilized. The TargetScan database predicted 23 miRNAs families broadly conserved among vertebrates. In case of the miRWalk database, it provided comparative analysis by 8 prediction programs and the number of miRNAs predicted by more than 3 prediction programs was 25. Among these miRNAs, 6 miRNAs were shared by the two groups (Fig. 2a). We transfected MSCs with those 6 miRNAs and examined the expression of HKII. Among these 6 miRNAs, miR-181a significantly decreased the expression of HKII in MSCs (Fig. 2b). To examine whether miR-181a directly targets HKII, we also conducted a luciferase assay. The luciferase assay used in the present study is to evaluate the effect of miRNA-dependent post-transcriptional regulation of target genes [14]. In this luciferase assay, 3′UTR of HKII was cloned into a plasmid containing the gene codes for luciferase. Since the 3′UTR of HKII was positioned immediately after the luciferase gene in the constructed plasmids, if miRNA effectively recognize and target the 3′UTR of HKII, the translation of luciferase is also affected and the expression of luciferase is suppressed. The result of the luciferase assay indicated that miR-181a transfection decreased the expression of luciferase linked to the 3′UTR of HKII (pmirGLO-HKII 3′UTR), while the luciferase expression of the cells transfected with control luciferase vector lacking the 3′UTR of HKII (pmirGLO) was not affected by miR-181a transfection. This indicated that miR-181a directly targeted the 3′UTR of HKII (Fig. 2c). To examine the possible relationship between oxidative stress and miR-181a, the expression of miR-181a in MSCs treated with H_2_O_2_ was evaluated. According to the real time PCR data, H_2_O_2_ significantly up-regulated the expression of miR-181a in a time-dependent manner (Fig. 2d).

### Neutralization of miR-181a attenuates cell death of MSCs induced by H_2_O_2_

**Fig. 3:**
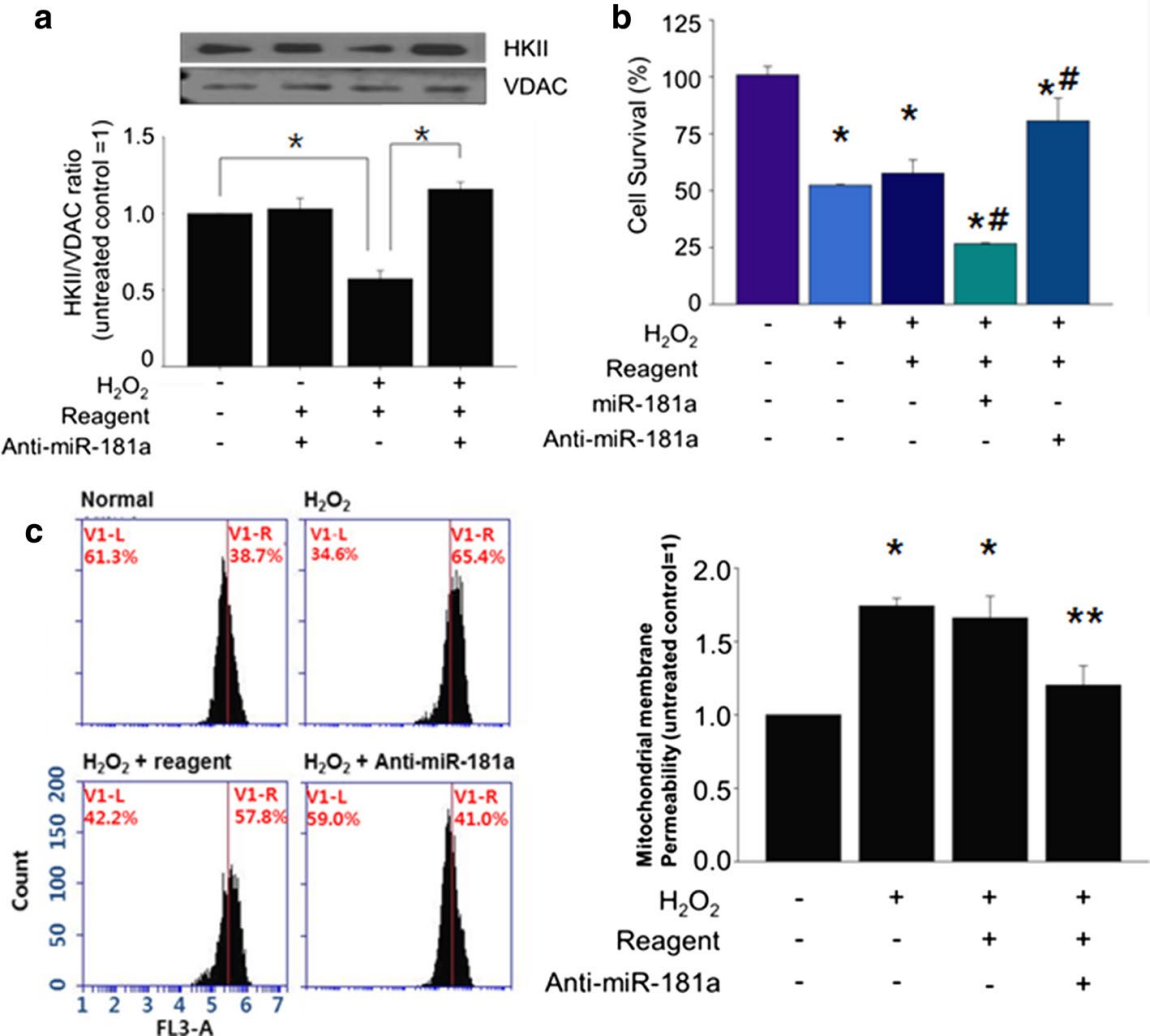
Anti-miR-181a attenuates HKII expression and H_2_O_2_-induced cell death of MSCs. **a** Effect of anti-miR-181a on HKII expression was examined by western blot. *p < 0.05. **b** Effect of anti-miR-181a on the survival of MSCs exposed to H_2_O_2_ was evaluated. *p < 0.05 compared to untreated control. ^#^p < 0.05 compared to H_2_O_2_ only treated group. **c** Effect of anti-miR-181a on H_2_O_2_-induced mitochondrial membrane potential disturbance was examined by using cytometry with TMRM. *p < 0.05 compared to untreated control. **p < 0.05 compared to H_2_O_2_ only treated group. The quantitative data were expressed as the mean ± s.e.m of at least three independent experiments.

Since we speculate that H_2_O_2_-induced up-regulation of miR-181a contributed to the down-regulation of mitochondrial HKII expression, we neutralized endogenous miR-181a using anti-miR-181a and examined its effect on mitochondrial HKII expression. Without H_2_O_2_ treatment, anti-miR-1861a transfection had no significant effect on mitochondrial HKII expression. However, with H_2_O_2_ treatment, anti-miR-181a attenuated mitochondrial HKII expression compared to H_2_O_2_ only treated group (Fig. 3a). This suggested that miR-181a-dependent regulation of HKII may be negligible under normal (without H_2_O_2_) condition. For the effect of anti-miR-181a on the survival of MSCs exposed to H_2_O_2_, H_2_O_2_-induced decrease of MSC survival was further decreased by miR-181a treatment, while anti-miR-181a treatment reversed such decrease (Fig. 3b), indicating suppression of H_2_O_2_-induced increase of miR-181a may be an effective way to enhance cell survival. Furthermore, H_2_O_2_ induced significant accumulation of tetramethylrhodamine methyl esters (TMRM) in the inner membrane of mitochondria indicating loss of mitochondrial membrane potential. However, such increase of TMRM accumulation was attenuated by anti-miR-181a transfection prior to H_2_O_2_ treatment suggesting that anti-miR-181a suppressed H_2_O_2_-induced mitochondrial membrane potential loss (Fig. 3c).

## Discussion

One of the major problems of cell therapy is low survival rate of transplanted cells. Thus developing an effective strategy to enhance the survival of the transplanted cells is of importance. Here, we report that miRNA-dependent modulation of HKII improves the survival of MSCs exposed to ROS.

The anti-apoptotic activity of HKII is based on their interaction with mitochondria [15, 16]. HKII, having the hydrophobic mitochondrial binding domain in their N-terminus [16, 17], can bind to mitochondria in association with the voltage-dependent anion channel (VDAC) [18], constituting the mitochondria permeability transition pore (PT-pore) [19]. This mitochondrial localization of HKII maintains mitochondrial integrity and prevent bcl-2 homologous antagonist killer (BAK) and bcl-2 associated protein X (BAX) mediated mitochondrial cytochrome c release and subsequent apoptosis [20, 21]. Our data also suggested that ROS-induced cell death via dissociation of HKII from mitochondria in our experimental settings.

H_2_O_2_-induced up-regulation of miR-181a has been also reported in rat cardiomyocytes [22]. That particular study clearly demonstrated that H_2_O_2_-induced up-regulation of miR-181a significantly contributed to apoptosis of cardiomyocytes. Thus, our premise that the cell death mechanisms of transplanted cells may be similar to those of host cells was supported. Although the above mentioned study did not examined the relationship between the up-regulation of miR-181a and HKII expression, it is possible that miR-181a-mediated down-regulation of HKII also contributed to the observed cell death of cardiomyocytes in that previous study.

To date, only few miRNAs that regulate HKII have been identified in mostly cancer cells. For example, miR-143 has been reported to down-regulate HKII in colon cancer cells, reducing cell proliferation [11]. Other studies also have demonstrated that miR-143 significantly increased the number of apoptotic cells compared to control in cancer cells [10, 12], suggesting down-regulation of HKII significantly contributes to progression of apoptosis. On the other hand, miR-143 repressive miR-155 led to up-regulation of HKII and promoted breast cancer cell proliferation [23]. These observations together suggest that miRNA-mediated regulation of HKII does occur, and modulation of HKII-targeting miRNAs to control cell survival is a feasible therapeutic approach.

The approach used in the present study was not intended to increase the mitochondrial binding efficacy of HKII, but to attenuate H_2_O_2_-induced increase of miR-181a and subsequent decrease of HKII expression. Although our data indicate that this approach works in vitro, it should be further validated in vivo as well with optimized delivery method of anti-miR-181a. Although MSCs were primed by anti-miR-181a treatment before exposed to H_2_O_2_ in this study, simultaneous delivery of primed MSCs and anti-miR-181a to damaged heart can be therapeutically more effective because anti-miR-181a delivery to damaged heart may enhance the survival of host cardiomyocytes on the verge of activation of apoptotic signaling as well. Another limitation of this study is lacking information about the effect of miR-181a modulation on the differentiation potential of MSCs. Since this study was focused on testing the feasibility of enhancing MSC survival by modulating key miRNA and its target, we have not examined the effect of anti-miR-181a on the differentiation of MSCs. Since previous studies have indicated that miR-181a accelerated endothelial cell [24] and adipocyte [25] differentiation of embryonic stem cells and pre-adipocyte, respectively, it can be speculated that suppressing miR-181a may alter the differentiation potential of MSCs as well. Nevertheless, it should be empirically proved, and examining such possibility in further studies will help us to develop improved regenerative therapeutic strategies involving MSC-based cell therapy.

## Conclusions

In summary, we demonstrate that anti-miR-181a attenuates cell death of MSCs exposed to H_2_O_2_ through suppression of miR-181a and subsequent down-regulation of HKII. With further optimization of delivery strategy and in vivo validation, anti-miR-181a can be an effective therapeutic agent for improving the survival of transplanted cells in cell therapy, and the result of this study warrants further studies to investigate more detailed underlying mechanisms.

## Methods

### Animals

Four-week-old Sprague–Dawley rats were used for MSC isolation. The rats were anesthetized with zoletil (20 mg/kg) and xylazine (5 mg/kg). All animal experimental procedures were approved by the Institutional Animal Care and Use Committee (IACUC) of Yonsei University College of Medicine and performed in accordance with the Guidelines and Regulations for Animal Care.

### Isolation and culture of rat MSCs

Bone marrow-derived MSCs were isolated and collected from aspirates of rat femurs and tibias with 10 mL of MSC medium consisting of Dulbecco’s modified Eagle’s medium (DMEM)-low glucose supplemented with 10 % fetal bovine serum (FBS) (Invitrogen) and 1 % antibiotic-penicillin and streptomycin. Mononuclear cells recovered from the interface of Ficoll-Paque PLUS (GE healthcare)-separated bone marrow were washed twice and resuspended in DMEM with 10 % FBS, then plated at a density of 1 × 10^6^ cells/100 mm dish. The cultures were maintained at 37 °C in a humidified atmosphere containing 5 % CO_2_. After 72 h, the non-adherent cells were discarded, and the adherent cells were thoroughly washed twice with phosphate-buffered saline (PBS). Fresh MesenPRO RS™ Medium (Invitrogen) was added and then replaced every 3 days for approximately 10 days to achieve stable multi-potentiality.

### H_2_O_2_ treatment

The cells were cultured for 24 h before the experiments. For inducing apoptosis, H_2_O_2_ was added to the culture media at a final concentration of 500–800 μM for 6 h.

### Cell viability assay

To measure cell viability, CCK reagent (cell counting kit-8, Dojindo) was added to each well for a final concentration of 0.5 mg/mL and the cells were incubated at 37 °C for 2 h. The absorbance of the medium was measured at 450 nm using a microplate reader.

### Transfection of miRNA and anti-miRNA

Transfections of miRNA mimics and anti-miRNAs were performed using siLentFect™ Lipid reagent (Life Science Research). Mature specific miRNAs (Genolution Pharmaceuticals, Inc., Korea) were used at a final concentration of 100 nM. Anti-miRNAs was used at a final concentration of 50 nM. After 4 h of incubation in a CO2 incubator at 37 °C, the medium was changed to 10 % FBS containing DMEM.

### Western blot

Cells were washed once in PBS and lysed in lysis buffer (Cell Signaling Technology) with protease and phosphatase inhibitor cocktail. Protein concentrations were determined using the BCA protein assay kit (Thermo Science). Proteins were separated in a SDS–polyacrylamide gel and transferred to the PVDF membrane (Millipore). After blocking the membrane with 0.1 % Tris-buffered saline–Tween 20 (TBS-T, 0.1 % Tween 20) containing 10 % skim milk for 1 h at room temperature, the membrane was washed twice with TBS and incubated with primary antibodies for overnight at 4 °C. The membrane was washed three times with 0.1 %TBS-T for 5 min and then incubated for 1 h at room temperature with horseradish peroxidase (HRP)-conjugated secondary antibodies. After extensive washing, the bands were detected by enhanced chemiluminescence reagent (ECL, Santa Cruz Biotechnology). The band intensities were quantified using NIH Image J version 1.34e software. The primary antibodies for HKII and β-actin were from Cell Signaling (28675) and Sigma (A1978), respectively.

### Luciferase assay using the 3′UTR of HKII

The 3′UTR sequences of HKII was amplified using primers with XbaI (forward) and EcoRI (reverse) endonuclease sites. The 3′UTR fragment was then cloned into the pmirGLO vector. HeLa cells were plated at a density of 1 × 10^5^ cell/well in a 12 well plate, and then transfected with either pmirGLO control vector or pmirGLO vector with HKII 3′UTR using Lipofectamine 2000. After 48 h, relative luciferase activity was measured by using Dual Luciferase assay kit (Promega) according to the manufacturer’s instructions. The Renilla luciferase was used for normalization.

### Real-time PCR

Purified total RNA (100 ng) was used for reverse transcription (TaqMan^®^ MicroRNA Reverse Transcriptase Kit, Applied Biosystems) in combination with TaqMan^®^ MicroRNA assays to quantify the levels of each specific miRNA according to the manufacturer’s instructions. The amplification and detection of specific products were performed using a TaqMan Small RNA Assay kit (AB Applied Biosystems) at 95 °C for 10 min, followed by 40 cycles of 95 °C for 15 s and 60 °C for 60 s. The threshold cycle (Ct) of each target gene was defined automatically; the Ct value was located in the linear amplification phase of the PCR and was normalized to the Ct value of the control transcript U6 (ΔCt value). The relative expression level of each miRNA was calculated using the ΔΔCt method and is reported as the fold-change in expression (2-ΔΔCt).

### Tetramethylrhodamine methyl ester (TMRM) staining

Mitochondrial membrane potential was determined by using tetramethylrhodamine methyl esters (TMRM) fluorescent dye (Invitrogen) [26]. Loss of mitochondrial membrane potential triggers the accumulation of cationic dye (TMRM) in the mitochondria. To evaluate mitochondrial membrane permeability, the cells were loaded with 200 nM of TMRM for 30 min at 37 °C in medium. Then, the cell pellets were washed with pre-warmed with PBS and re-suspended with PBS to analyze. The data were performed by BD ACCURI C6 cytometer (BD Biosciences) and analyzed with BD Accuri C6 Software.

### Statistical analysis

Quantitative data were expressed as the mean ± S.E.M (standard error of measurement) of at least 3 independent experiments. For statistical analysis, one-way ANOVA with Bonferroni correction was performed using the OriginPro 8 SR4 software (ver. 8.0951, OriginLab Corporation, Northampton, MA, USA). A *p* value of less than 0.05 was considered to be statistically significant.
